# Manoeuvre Target Tracking in Wireless Sensor Networks Using Convolutional Bi-Directional Long Short-Term Memory Neural Networks and Extended Kalman Filtering

**DOI:** 10.3390/s24134261

**Published:** 2024-06-30

**Authors:** Duo Peng, Kun Xie, Mingshuo Liu

**Affiliations:** School of Computer and Communication, Lanzhou University of Technology, Lanzhou 730050, China; 222085402003@lut.edu.cn (K.X.); 222085402007@lut.edu.cn (M.L.)

**Keywords:** mobile target tracking, convolutional long short-term neural networks, extended Kalman filtering, wireless sensor networks, received signal strength

## Abstract

Aiming at the problem that traditional wireless sensor networks produce errors in the positioning and tracking of motorised targets due to noise interference, this paper proposes a motorised target tracking method with a convolutional bi-directional long and short-term memory neural network and extended Kalman filtering, which is trained by using the simulated RSSI value and the actual target value of motorised targets collected from the convolutional bi-directional neural network to the sensor anchor node, so as to obtain a more accurate initial value of the manoeuvre target, and at the same time, the extended Kalman filtering method is used to accurately locate and track the real-time target, so as to obtain the accurate positioning and tracking information of the real-time target. Through experimental simulation, it can be seen that the algorithm proposed in this paper has good tracking effect in both linear manoeuvre target tracking scenarios and non-linear manoeuvre target tracking scenarios.

## 1. Introduction

Wireless Sensor Networks (WSNs), as the cornerstone of modern IoT technology, build an efficient and intelligent data collection and processing network through the extensive deployment of wireless sensor devices. These sensors are capable of detecting and integrating various data in the environment in real time, which greatly enriches the data sources of IoT and provides valuable support for various application scenarios. Especially in the field of target tracking, WSNs show their unrivalled advantages and potential.

Target tracking, as one of the important applications of WSN technology, has demonstrated its great value in many fields such as traffic monitoring and war detection. By arranging wireless sensors in the environment where the target is likely to appear, WSN is able to capture real-time measurements of the target’s movement, such as speed and direction. Subsequently, combined with advanced tracking system control strategies, WSNs are able to accurately estimate the position of the tracked target, thus realizing precise tracking of the target [1]. This technique not only provides real-time position information, but also predicts the future trajectory of the target’s motion, providing strong support for decision making.

However, to achieve accurate target localization and tracking, WSNs face many challenges. Among them, determining the location of mobile nodes is a particularly critical task. Although equipping each node with a GPS or BDS module is the most straightforward approach, it is not practical given the cost, energy consumption, and deployment environment constraints. Therefore, researchers usually use other nodes to estimate the distance to the anchor node through ranging or connectivity information, and then use maximum likelihood estimation, triangulation, and other methods to calculate the node’s position.

In WSNs, tracking algorithms are the core of achieving accurate target localization and tracking. These algorithms mainly include localization algorithms and filter prediction algorithms [2]. The localization algorithms can be further categorized into distance measurement-based algorithms and distance measurement-free algorithms. Distance measurement-based algorithms determine coordinates by measuring the distance and angle between nodes, which is highly accurate but costly and consumes a lot of power. The distance measurement-free algorithms, on the other hand, estimate the position through communication and collaboration between nodes, which reduces the cost and power consumption, but the positioning accuracy is relatively low.

In terms of filter prediction algorithms, algorithms such as Kalman filter and particle filter play an important role in target tracking. In particular, Extended Kalman Filtering (EKF) performs well in dealing with nonlinear and non-Gaussian problems because it is not limited by linear and Gaussian noise distributions. EKF is able to achieve accurate tracking of dynamic targets by recursively predicting and updating the target state. However, the performance of EKF is limited by the discrepancy between the noise model and the actual environment, and thus needs to be adapted and optimized according to the do situation in practical applications.

Another key challenge in realizing precise target localization and tracking in WSNs is to improve the accuracy of initial position estimation as well as the ranging accuracy of dynamic, real-time targets. Received Signal Strength Indication (RSSI) is a commonly used technique for estimating the distance between nodes based on signal strength in WSNs. However, RSSI measurements are often affected by factors such as multipath effects, non-line-of-sight (NLOS) propagation, and hardware variations, which lead to measurement errors [3]. To overcome these challenges, researchers have proposed a series of innovative algorithms and techniques, such as RSSI-based fingerprint localization algorithms and machine learning-based ranging correction methods. These methods improve the accuracy of ranging and initial position estimation by combining multi-source information and optimizing algorithm parameters to achieve more accurate target tracking.

Although scholars have proposed many innovative algorithms and techniques for manoeuvring target tracking in WSNs, each method has its limitations. For example, the traditional method combining RSSI measurements with recursive Bayesian frame filters performs well in initial position estimation, but its localization limits the improvement of global tracking accuracy. Global filtering methods, while capable of solving the initial position estimation problem, face significant challenges in tracking dynamic targets. While Kalman filtering (KF) and untracked Kalman filtering (UKF) methods are able to deal with the noise problem in dynamic target tracking, their performance is limited by the discrepancy between the noise model and the actual environment, and UKF requires a large number of sensors, which increases the complexity and cost of the system [4].

This study aims to address these limitations by introducing a novel target tracking algorithm for WSNs that combines a Convolutional Bi-directional Long Short-Term Memory (Conv-BiLSTM) network and Extended Kalman Filtering (EKF) techniques. The proposed algorithm extracts spatio-temporal features of the target using Conv-BiLSTM and iteratively updates the dynamic system using EKF. This approach aims to improve the accuracy, real-time performance, and robustness of target tracking, especially when dealing with nonlinear systems and uncertainty. By integrating these techniques, we expect to overcome the limitations of existing approaches and provide a cost-effective solution for efficient target tracking in WSNs [5].

## 2. Related Work

In the field of mobile target tracking for wireless sensor networks (WSNs), a great deal of research has been conducted by scholars at home and abroad, and these studies have made significant progress in terms of target localisation, tracking accuracy, and real-time performance. However, although several methods have been proposed, each method has certain limitations. Beborta [5] has achieved significant results in target initial position estimation by combining RSSI measurements with recursive Bayesian frame filters. However, RSSI measurements are susceptible to environmental factors, resulting in limited positioning accuracy, and their localised characteristics limit further improvements in global tracking accuracy. The global filtering method proposed in [6,7,8] performs well compared to the traditional filtering algorithms in target initial position estimation. However, when confronted with dynamic targets, the performance of these methods in continuous tracking is limited as they may not be able to adapt to changes in the target state in a timely manner. Zhang, Y. [9] used Kalman filtering (KF) to analyse the noise problem in dynamic target tracking and improved it accordingly. However, Kalman filtering has more stringent assumptions regarding the noise model, and its tracking performance will be significantly affected when the actual noise differs significantly from the assumed model. Li, S. [10] used untracked Kalman filtering (UKF) to track the position and velocity of the target, which is based on the dynamic model of human walking and can improve the real-time tracking and accuracy to a certain extent. However, the high complexity and cost requirements of untraceable Kalman filtering for large-scale sensor networks limit its wide application in WSNs. Although the single-sensor distance sensing method proposed in Bu, D. [11] is simple and fast to implement, its measurement error is large due to its reliance on only a single sensor, which makes it difficult to meet the application requirements.

Extended Kalman Filtering has significant advantages in dealing with nonlinear systems. It effectively estimates the system state by performing local linearization near the dynamic and measurement models of nonlinear systems. This type of filter can process measurement data containing noise in real time and efficiently; even when there is a nonlinear relationship between the system dynamics or measurement model, it can provide accurate state estimation. Manoeuvring target tracking is a system that requires fast response, and the extended Kalman filter has a faster iteration speed compared to other methods. Therefore, the extended Kalman filter algorithm is selected in this paper.

The proposed motorised target tracking algorithm, which combines convolutional bi-directional long- and short-term memory neural networks and extended Kalman filtering, offers significant advantages and distinguishing features compared to existing methods in the context of wireless sensor networks.

Firstly, the integration of Conv-BiLSTM neural networks enables the algorithm to accurately model the complex, nonlinear relationship between RSSI (Received Signal Strength Indication) values and target distances. Conv-BiLSTM’s bi-directional structure allows it to capture temporal dependencies in both forward and backward directions, while its convolutional layers extract local features from RSSI data, providing a richer input for the LSTM layers. This results in a more precise mapping between RSSI and target distance, leading to improved initial position estimation and overall tracking accuracy.

Secondly, the introduction of extended Kalman filtering addresses the uncertainty introduced by zero-mean Gaussian measurement noise and sudden changes in target speed. Extended Kalman filtering is particularly suited for nonlinear systems, and it effectively fuses the predictions from the Conv-BiLSTM model with new sensor measurements to provide an optimal estimate of the target’s state. This helps achieve real-time, two-dimensional tracking of a single motorised target with improved robustness and accuracy.

Thirdly, the combined algorithm reduces the absolute error of motorised target tracking and localisation. By accurately modelling the RSSI–distance relationship and effectively handling measurement noise and target speed changes, the proposed method achieves more precise target tracking and positioning. This is crucial for applications that require high-accuracy target tracking, such as surveillance, autonomous navigation, and industrial automation.

Finally, the algorithm also reduces computational overhead to a certain extent. While Conv-BiLSTM networks are more complex than traditional linear models, their efficient implementation and the use of extended Kalman filtering help to optimize the overall computational cost. This ensures that the algorithm can be executed in real-time, making it suitable for resource-constrained WSN environments.

## 3. Manoeuvre Target Tracking Using Convolutional Bi-Directional Long- and Short-Term Memory Neural Networks and Extended Kalman Filtering

**Figure 1:**
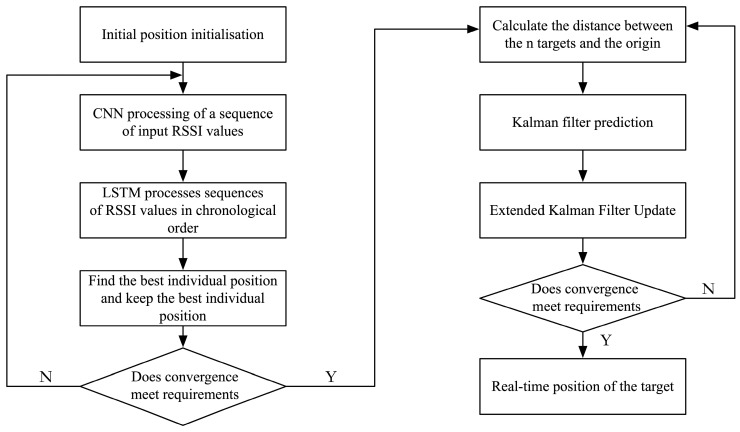
Algorithm flowchart.

The method in this paper is trained using a convolutional neural network using the received signal strength indication (RSSI) value and the actual target 2D position to obtain the accurate initial position of a single manoeuvre target during 2D motion. Then, the real-time target is accurately positioned and tracked using the extended Kalman filter to obtain accurate positioning and tracking information of the real-time target. The algorithm flowchart proposed in this article is shown in Figure 1.

This paper presents a novel method that combines Received Signal Strength (RSSI) values, Convolutional Neural Networks (CNNs) and Extended Kalman Filtering (EKF) for initial position localisation and real-time tracking of a single manoeuvrable target in 2D space. The method is first trained by collecting the RSSI values of the target and its corresponding 2D position information using a CNN in order to learn the mapping relationship from the RSSI values to the initial position of the target. This approach makes full use of the correlation between the RSSI values and the target position to significantly improve the accuracy of the initial position estimation.

In the training phase, the CNN model learns from a large amount of labelled data (RSSI values and corresponding target positions) and is thus able to accurately predict the initial position of the target for a given RSSI value. This data-driven approach overcomes the reliance on complex physical models in traditional methods and improves the robustness and generalisation of the system.

Once the initial position estimate of the target is obtained, the system turns to the EKF for real-time accurate target localisation and tracking. The EKF is capable of predicting and updating the state of the target by continuously fusing new observations and the dynamic model of the target, thus achieving continuous and accurate tracking of the target. This method is not only able to cope with the dynamic changes of the target, but also can effectively deal with the measurement noise and model error, which improves the accuracy and stability of tracking.

Regarding RSSI, it is an indication of the signal strength received during wireless signal transmission. In wireless sensor networks, there is a correlation between the RSSI value and the distance to the target, so the distance between the target and the sensor can be estimated by measuring the RSSI value. However, due to environmental factors, the relationship between RSSI values and distances is often nonlinear, and thus methods such as machine learning are needed to establish accurate mapping relationships. The method proposed in this paper is based on this idea, and uses CNN to learn this nonlinear relationship from RSSI values, so as to achieve the accurate positioning of the target. It is used to study the initial position localization and real-time tracking of a single manoeuvring target in two-dimensional space. The simulation model of the system consists of a set of static anchor nodes with known coordinates that are deployed in a 100 m × 100 m simulation area. It is assumed that the moving target carries a wireless sensor network (WSN) node that is capable of receiving wireless signals sent by all anchor nodes at each time step t. The wireless signals are transmitted from all anchor nodes at each time step.

At each time step, the Received Signal Strength Indication (RSSI) values collected from all anchor nodes are sent to an external base station. The key assumption here is that the mobile target acts as a transceiver and is able to receive signals from anchor nodes and measure their RSSI values, which are then sent to an external base station for further processing. On the other hand, the anchor node acts as a transmitter with an all-isotropic antenna that ensures that the wireless signal covers the simulation area uniformly.

This system architecture allows us to test and analyse the performance of the target localization and tracking algorithm proposed in this paper by simulating the propagation and reception of wireless signals, which combines CNN and EKF. By adjusting the number and location of the anchor nodes, as well as the propagation parameters of the wireless signals, we can simulate different indoor or outdoor environments to evaluate the accuracy and robustness of the algorithm under different conditions. In this paper, the simulation conditions are based on outdoor conditions, and the propagation model chosen is the logarithmic distance path loss model with *n* value set to 3. The logarithmic distance path loss model is shown as follows:

(1) $$P\left(d\right)=P(d_{0})-10n\mathrm{lg}(\frac{d}{d_{0}})+X\sigma$$

In the formula, *P*(*d*) is the received signal strength at the reference distance. Generally, *P*(*d*_0_) takes a value of 1, representing the path propagation loss after propagating 1 m. n is the path loss index, *Xσ* is a random variable that represents the influence of other factors on signal strength.

In summary, the method proposed in this paper combines the advantages of CNN and EKF, and achieves initial position localisation and real-time tracking of a single manoeuvre target by exploiting the correlation between the RSSI value and the target position. Compared with existing methods, the method has significant advantages in terms of positioning accuracy, robustness, and real-time performance. 

### 3.1. Neural Network Accuracy Testing

**Figure 2:**
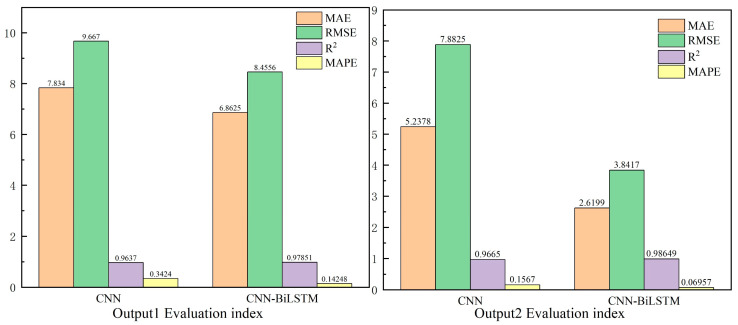
Comparison of prediction results of different models.

In order to assess the prediction accuracy of the TDOA/FDOA correction model combining CNN–BiLSTM, two models, CNN and CNN-BiLSTM, were trained separately for experimental simulation. The prediction models proposed in this paper will be evaluated for their effectiveness using the following metrics: the Mean Absolute Error (*MAE*), Root Mean Square Error (*RMSE*), Mean Absolute Percentage Error (*MAPE*), and Coefficient of Determination (*R*^2^) as the evaluation index to measure the prediction effect. Mean Absolute Percentage error (*MAPE*) and These are calculated as shown in Equation (2):

(2) $$\left\{\begin{array}{l} MAE=\frac{1}{n}\sum_{i=1}^{n}\left|y_{i}-p_{i}\right|\\ RMSE=\sqrt{\frac{1}{n}\sum_{i=1}^{n}{\left(y_{i}-p_{i}\right)}^{2}}\\ MAPE=\frac{1}{n}\sum_{i=1}^{n}\left|\frac{y_{i}-p_{i}}{y_{i}}\right|\\ R^{2}=1-\frac{\sum_{i=1}^{n}{\left(y_{i}-p_{i}\right)}^{2}}{{\sum_{i=1}^{n}\left(y_{i}-\bar{y}\right)}^{2}} \end{array}\right.$$

where: *n* is the number of test sets; *i* is the actual TDOA/FDOA value; *y_i_* is the predicted TDOA/FDOA value; and *p_i_* is the average value of the actual data. The simulation results are shown in Figure 2.

The simulation comparison between Output1 and Output2 shows that the evaluation metrics of MAE, RMSE, and MAPE of the proposed improved prediction method are less than those of the CNN model. This indicates that the prediction accuracy of the proposed model is higher than the other two algorithms. In addition, the value of the parameter for Output1 is 0.978, while the value of the parameter for Output2 is 0.986. The parameter is mainly used to assess the fit of the model, and a value close to 1 indicates a better quality of the model. Therefore, this verifies that the proposed algorithm outperforms the other algorithms in terms of prediction accuracy and model quality.

### 3.2. Tracking Effectiveness Test

#### 3.2.1. Tracking System Modelling

In order to verify that the proposed algorithm has good tracking performance under both a linear manoeuvre model and a nonlinear manoeuvre model, the simulation experiments in this paper use the uniform turn model and variable acceleration linear model to verify the advantages of the algorithm in this paper, respectively, and the equations of motion state of the uniform turn motion model are as follows:

(3) $$F=\left(\begin{array}{cccc} 1 & \frac{\sin\Omega \Delta t}{\Omega } & 0 & \frac{1-\cos\Omega \Delta t}{\Omega }\\ 0 & \cos\Omega \Delta t & 0 & -\sin\Omega \Delta t\\ 0 & \frac{1-\cos\Omega \Delta t}{\Omega } & 0 & \frac{\sin\Omega \Delta t}{\Omega }\\ 0 & \sin\Omega \Delta t & 0 & \cos\Omega \Delta t \end{array}\right)$$

(4) $$G_{k}=\left(\begin{array}{cc} \frac{\Delta t_{k}^{2}}{2} & 0\\ \Delta t_{k} & 0\\ 0 & \frac{\Delta t_{k}^{2}}{2}\\ 0 & \Delta t_{k} \end{array}\right)$$

(5) $$x_{k+1}=Fx_{k}+Gw_{k}$$

where *w_k_* is the system noise in the equation of state.

The equation of state of motion for the uniformly variable linear motion model is shown below:

(6) $$F=\left[\begin{array}{cccccc} 1 & T & \frac{1}{2}T^{2} & 0 & 0 & 0\\ 0 & 1 & T & 0 & 0 & 0\\ 0 & 0 & 1 & 0 & 0 & 0\\ 0 & 0 & 0 & 1 & T & \frac{1}{2}T^{2}\\ 0 & 0 & 0 & 0 & 1 & T\\ 0 & 0 & 0 & 0 & 0 & 1 \end{array}\right]$$

where *T* is the sampling interval.

#### 3.2.2. WSN Ranging Model Modelling

For the WSN intra-area tracking problem, it is assumed that the sensors in the sensor network are all of the same model and the sensors use the following observation model:

(7) $$Z_{g}=\sqrt{X_{g}^{2}+Y_{g}^{2}}+v_{g}$$

where *v_g_* is the system noise and measurement noise.

(8) $$z_{i}=\left(1+y_{i}\right)r_{i}+n_{i}=r_{i}+u_{i}$$

(9) $$r_{i}=\sqrt{{\left(x-x_{i}\right)}^{2}+{\left(y-y^{i}\right)}^{2}}$$

where (*x*, *y*) is the position of the target at moment *t*, (*x_i_*, *y_i_*) is the position of sensor *i*, and u_i_ is the random noise generated with covariance Q.

#### 3.2.3. Simulation Condition

Twenty sensors are randomly arranged in a 100 m × 100 m wireless sensor network monitoring area, the communication radius of the sensors is set to 30 m, the sampling period is set to 0.1 s, and the number of sampling points is set to 50. In the filtering algorithm, the filtered ambient noise is set to diag([0.5,0.5]), and the filtered observation noise is set to 1 [12,13,14,15].

In the simulation experiments, *RMSE* is used as a measure of tracking effectiveness with the following equation:

(10) $$RMSE=\sqrt{\frac{1}{N}\sum_{i=1}^{N}\sqrt{{\left(\tilde{x}-x\right)}^{2}+{\left(\tilde{y}-y\right)}^{2}}}$$

where *RMSE* is the normalised mean positioning error of the node, $\left(\hat{x},\hat{y}\right)$ is the predicted coordinates at time *t*, and $\left(x,y\right)$ is the actual unknown node coordinates.

#### 3.2.4. Simulation Results

After establishing the target model through the above process and setting the initial conditions of the simulation, the trajectory is tracked using the algorithm of this paper. In order to verify the effectiveness of this paper’s algorithm, 100 repetitive experiments were carried out on manoeuvre target tracking using the PF algorithm, EKF algorithm, RSSI algorithm, and the corresponding algorithm in the literature [16], and RMSE (Root Mean Squared Error) was used as a criterion for evaluating the algorithm’s performance.

(1) For the uniform turning motion model

The initial value of the x-direction of the trajectory is set at 10 m, the initial value of the y-direction is set at 10 m, and the initial value of the velocity in the x-direction and y-direction is set to 5 m/s, and acceleration is set to 0.122 [17,18,19,20,21,22].

**Figure 3:**
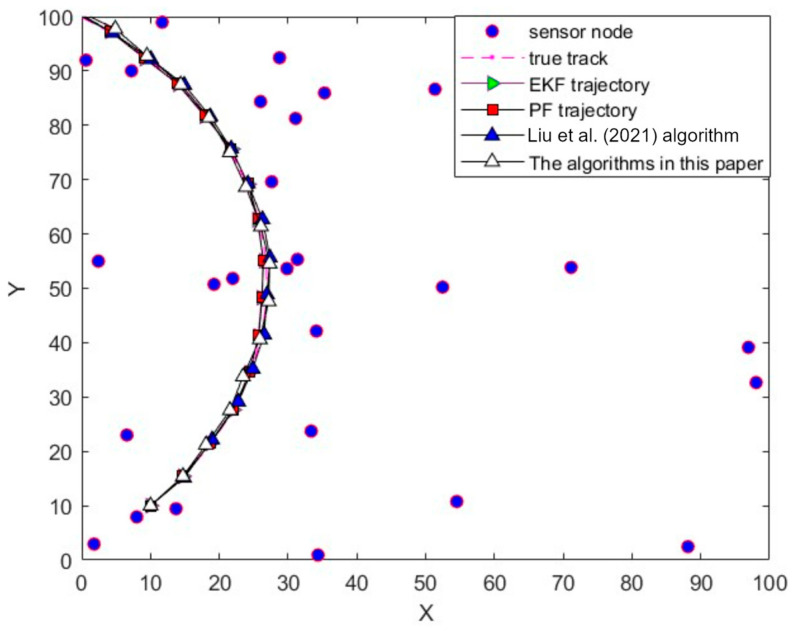
Trajectory comparison; [16]—Liu et al. (2021).

The two-dimensional spatial trajectory comparison of the target tracking trajectory is shown in Figure 3. From Figure 3 and Table 1, it can be seen that the tracking trajectory of this paper’s algorithm and the real trajectory almost overlap, and the root mean square error of the tracking is only 0.2133, which shows that using this paper’s algorithm to track the target of the nonlinear model is effective.Table 1 is a schematic diagram of tracking error, and Figure 1 is a schematic diagram of tracking effect.

**Figure 4:**
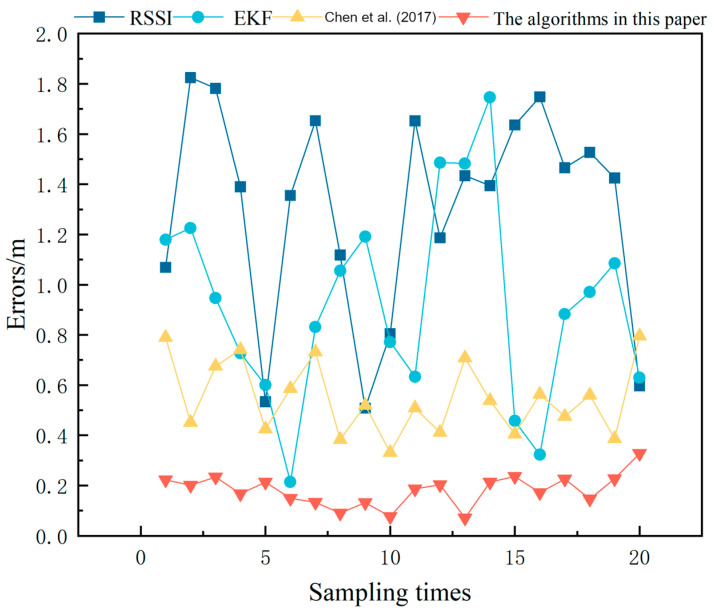
Error comparison; [18]—Chen et al. (2017).

Figure 4 shows the position error comparison of the three algorithms, and it can be seen from Table 1 and Figure 4 that, compared with the other algorithms, the average square root error of the position of this paper’s algorithm decreases compared with all of the above algorithms. It can be seen that the proposed convolutional bi-directional long- and short-term memory neural network and iterative Kalman filtering manoeuvre target tracking method in this paper can effectively correct the errors due to noise and sudden changes in speed, so as to predict and track the position of the target more accurately. Meanwhile, due to the addition of convolutional bi-directional long short-term memory neural network, the algorithm proposed in this paper is more stable in tracking and the initial value estimation is more accurate compared with other algorithms.The schematic diagram of tracking error is shown in Figure 4.

**Figure 5:**
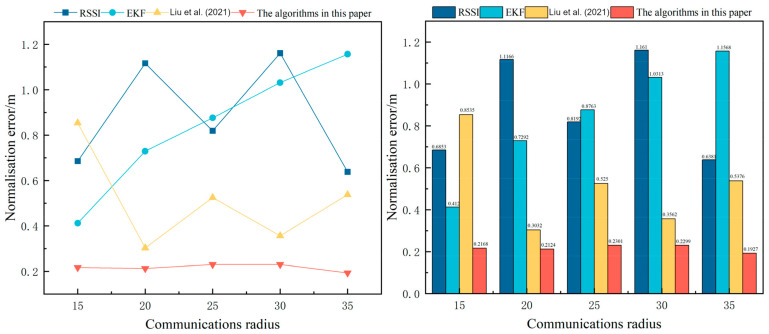
Effect of communication radius on tracking normalisation error; [16]—Liu et al. (2021).

Figure 5 shows the comparison of the effect of communication radius on the tracking root mean square error; from the figure, it can be seen that due to the powerful processing ability of CNN-BiLSTM, the reduction in communication radius will increase the path loss of the communication link, which makes the communication between the nodes weaker. At the same time, CNN-BiLSTM processing RSSI value will greatly reduce the communication loss between nodes, thus having a more stable tracking effect.The impact of communication radius on tracking error is shown in Figure 5.

**Figure 6:**
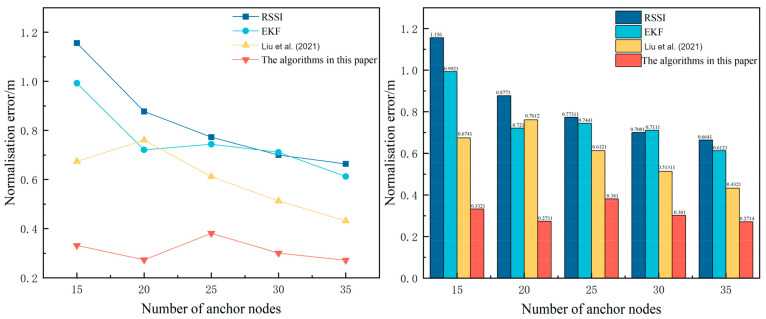
Effect of the number of anchor nodes on the normalisation error; [16]—Liu et al. (2021).

As shown in Figure 6, the root mean square error decreases with the increase in anchor nodes, and only a limited number of anchor nodes provide the collected tracking information to accurately determine the target location, while the algorithm proposed in this paper not only reduces the RSSI error by using the CNN-BiLSTM, but also adopts the Extended Kalman Filtering algorithm to greatly reduce the increase in estimation error due to the problem of collecting too little data. Experiments show that the algorithm proposed in this paper still has a low normalisation error when the sensor nodes are low.The impact of sensor node tree on tracking error is shown in Figure 5.

(2) For the uniformly accelerated linear motion model

In order to verify that the algorithm proposed in this paper still has a good tracking effect under a linear motion model, the uniformly accelerated motion model is selected. The initial value of the x-direction of the motorised target trajectory is 10 m, the initial value of the y-direction is 10 m, the initial value of the x-direction and y-direction speed is set to 5 m/s, and acceleration is set to 0.5 m/s.

**Figure 7:**
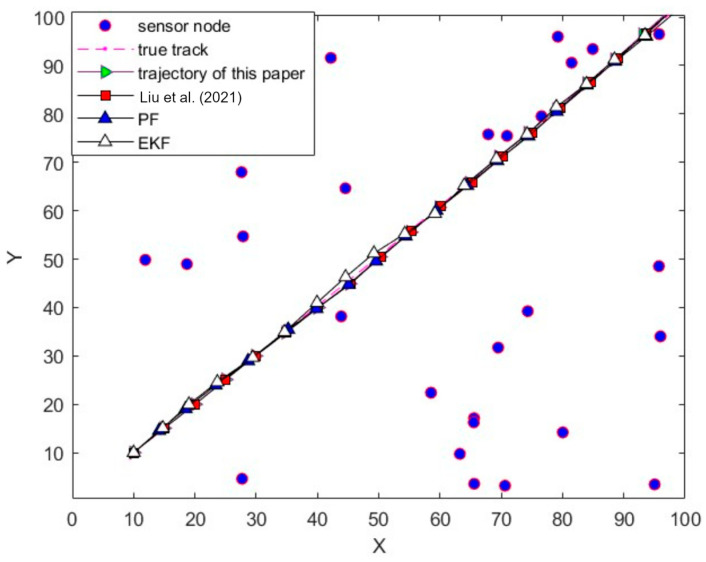
Trajectory comparison; [16]—Liu et al. (2021).

From Figure 7 and Table 2, it can be seen that the algorithm proposed in this paper has good tracking error in linear manoeuvre target tracking. In the experiments targeting the initial position determination of the manoeuvre target, the Convolutional Bi-directional Long Short-Term Memory Neural Network (CNN-BiLSTM) is employed to deal with the tracking problem in the context of zero-mean Gaussian measurement noise. The experimental results show that CNN-BiLSTM exhibits significant advantages in linear tracking systems.The error and schematic diagram of linear motion are shown in Table 1 and Figure 7, respectively.

CNN-BiLSTM effectively reduces the impact of Gaussian measurement noise on tracking results through its powerful learning and adaptation capabilities. By learning and recognising noise patterns in historical data, ConvLSTM is able to automatically adjust during the prediction process to improve the accuracy of tracking, while BiLSTM combines the advantages of convolutional neural networks (CNNs) and long short-term memory neural networks (LSTMs), enabling it to process both spatial and temporal information. In the 2D tracking problem, CNN-BiLSTM not only extracts the spatial features in the image by a CNN, but also captures the changes of these features in the time series by using an LSTM, which more accurately describes the motion state of the manoeuvring target. Further, the bi-directional temporal processing capability of CNN-BiLSTM enables it to consider both past and future information. This is particularly important for predicting the future position of a manoeuvre target, as the movement of a manoeuvre target is often influenced by its historical trajectory, its current state, and its future intentions or environment. By considering these factors simultaneously, CNN-BiLSTM is able to provide more accurate predictions.

In summary, the experimental analysis shows that using CNN-BiLSTM to determine the initial position of a manoeuvre target and applying it in a linear tracking system in the context of zero-mean Gaussian measurement noise can significantly improve the accuracy and precision of tracking. The noise robustness, spatial feature extraction capability, bi-directional temporal processing capability, and on-line learning capability of CNN-BiLSTM make it an effective tracking method for manoeuvring targets.

## 4. Conclusions

To address the problem that traditional wireless sensor networks are prone to errors in the positioning and tracking of moving targets due to noise interference, this paper proposes a motorised target tracking method with a convolutional bi-directional long- and short-term memory neural network and extended Kalman filtering, which uses a convolutional neural network to process a sequence of input RSSI values to obtain a more accurate estimation of the x-axis and y-axis coordinates of the mobile node at the current moment [22,23,24,25,26,27,28,29,30], and at the same time, uses the extended Kalman filtering method to accurately locate and track the real-time target, and obtain accurate positioning and tracking information of the real-time target. Through experimental simulation and comparison, it can be seen that the algorithm proposed in this paper can obtain good tracking effect for both linear and nonlinear targets.

Future research will focus on exploring how to reduce algorithm complexity while maintaining high positioning accuracy. This includes designing lightweight network structures, optimising feature extraction and fusion methods, applying model compression and acceleration techniques, investigating incremental learning and adaptive optimisation algorithms, and exploring distributed and cooperative target tracking strategies [31,32]. In addition, privacy protection and security as well as cross-domain fusion and applications will also be important directions for future research.

## Figures and Tables

**Table 1:** Tracking Error Comparison.

	Position RMSE (m)	Mean Value of Position Error in X Direction (m)	Mean Value of Position Error in Y Direction (m)
RSSI	1.2241	1.3911	0.8122
EKF	0.8012	0.5123	0.4022
PF	0.6421	0.4422	0.3251
Literature [18] algorithm	0.4133	0.3221	0.1234
The algorithm in this paper	0.2133	0.154	0.09122

**Table 2:** Comparison of tracking error.

	Position RMSE (m)	Mean Value of Position Error in X Direction (m)	Mean Value of Position Error in Y Direction (m)
RSSI	0.8812	0.6741	0.5712
EKF	0.7111	0.5123	0.4781
Literature [18] algorithm	0.5662	0.4212	0.2345
The algorithm in this paper	0.2023	0.1761	0.08144

## Data Availability

Data are contained within the article.
